# Developments in Quantum Probability and the Copenhagen Approach

**DOI:** 10.3390/e20060420

**Published:** 2018-05-31

**Authors:** Gregg Jaeger

**Affiliations:** 1 Quantum Communication and Measurement Laboratory, Department of Electrical and Computer Engineering, Boston University, Boston, MA 02215, USA; jaeger@bu.edu; 2 Division of Natural Science and Mathematics, Boston University, Boston, MA 02215, USA

**Keywords:** quantum probability, potentiality, complementarity, uncertainty relations, Copenhagen interpretation, indefiniteness, indeterminism, causation, randomness

## Abstract

In the Copenhagen approach to quantum mechanics as characterized by Heisenberg, probabilities relate to the statistics of measurement outcomes on ensembles of systems and to individual measurement events via the actualization of quantum potentiality. Here, brief summaries are given of a series of key results of different sorts that have been obtained since the final elements of the Copenhagen interpretation were offered and it was explicitly named so by Heisenberg—in particular, results from the investigation of the behavior of quantum probability since that time, the mid-1950s. This review shows that these developments have increased the value to physics of notions characterizing the approach which were previously either less precise or mainly symbolic in character, including complementarity, indeterminism, and unsharpness.

## 1. Introduction

The orthodox approach to quantum theory emerged primarily from interactions in Copenhagen and elsewhere from the work of Niels Bohr, Werner Heisenberg, and Wolfgang Pauli, depending also on contributions of Max Born, and was largely set out by 1927, cf. [1,2,3,4]. After various criticisms of the initial form, Bohr focused more strongly on complementarity in the 1930s, and Heisenberg—in a strong response of 1955 in which the basis of the approach can be considered to have been essentially finalized—added the new element of actualization of potentiality to its approach to quantum probability [5]. Since then, much attention has been paid to newly emerging alternative treatments of quantum physics, such as Bohmian mechanics and collapse-free (e.g., many-worlds) mechanics, that use quantum probability and the quantum formalism. Indeed, with a few exceptions (e.g., [2,6,7,8] and others mentioned below) relatively little attention has been paid to the implications of new research for the more orthodox, Copenhagen approach. Here, a non-exhaustive but wide-ranging series of theoretical results, several directly related to experiment, obtained since the work of Heisenberg related to quantum probability, is discussed, which, by articulating more precisely and better clarifying the application of its basic notions, including complementarity, uncertainty, and indeterminacy, which were before either less precise or even merely symbolic, further demonstrates the value of this approach.

Beyond the basic notion that the probability of any observed future physical event in a system can be found via the quantum state using the Born rule given the results of a complete set of measurements on it, the character of quantum probabilities on this Copenhagen approach is discussed in the next section; experimentally verified results demonstrating new quantum complementarities are considered in Section 3; theoretical developments involving unsharpness and quantum measurement are considered in Section 4; novel explications of indeterminism and randomness are considered in Section 5.

## 2. Probability in the Copenhagen Approach

The Copenhagen approach to quantum theory generally gives primacy to measured phenomena—with any results regarding the measured system being given in relation to the entire experimental situation in which each arises and with the records of measuring devices being classical describable—without being positivist and remaining essentially realist. Although Bohr’s notion of complementarity was the greatest influence early in the development of the approach—as noted, for example, by Jan Faye [9] and Arkady Plotnitsky [10,11]—it was later supplanted in a development toward more precise and mathematically advanced treatments of the effect, as exhibited in Section 3 (also cf. [12])—and even explicitly extended to situations involving entanglement as, arguably, thought by Bohr to be the case, as Don Howard has argued [13]. The Copenhagen approach explicitly named as such is that circumscribed by Werner Heisenberg [5] in which measurement corresponds to the actualization of a potential physical situation where a single value appears from among a set of possible values that were not certain (cf. [14,15] for analyses of the fundamentals of this version, specifically considered here). The essential mathematical formalism of non-relativistic QM emerged with work of Paul Dirac and John von Neumann, with Hilbert-space as the space of individual system states (cf. [1,2,3] and Chapter. 7 in [16]), forming the context for its later extension, discussed in detail below, cf. [2,14,17].

A succinct overview of the role of probability in the Copenhagen interpretation was given by Heisenberg, who gave the interpretation its name: “…the probability function does not in itself represent a course of events in the course of time. It represents a tendency for events and our knowledge of events. The probability function can be connected with reality only if one essential condition is fulfilled: if a new measurement is made to determine a certain property of the system. Only then does the probability function allow us to calculate the probable result of the new measurement” (pp. 46–47, [18] ; cf. [15]).

### 2.1. Quantum States and Probability

An (O,S,p) formulation of general physical theory serves as a basic formal framework for the non-relativistic theory of QM [19,20]: To each physical system, one can associate with the set of all associated sharp observables (Hermitian self-adjoint operators) *O* and the set of its states *S*, a function *p*: O×S×B(R)→[0,1], where B(R) is the set of all Borel subsets of R, cf. [21]—of values appearing in measurements, cf. [2]. Restricting ourselves specifically to the Hilbert-space formulation of quantum mechanics, each statistical operator ρ is decomposable into a non-trivial weighted sum of quantum pure states represented by normalized vectors ${|\psi \rangle }_{i}\in H$ (cf. [5,18]) as $\rho_{i}=|\psi_{i}\rangle \langle \psi_{i}|$ that have no further state decomposition; each statistical operator ρ also induces an expectation functional A↦tr(ρA) on L(H), the space of linear operators on the Hilbert space H.

The probability *p* in the Copenhagen approach involves an explicit distinction between objective and subjective aspects of physical states describable in this formalism and compares with that in classical mechanics as follows. In classical mechanics, when needed at all, probability is used only in situations where a detailed knowledge of the system is lacking, i.e., for statistical mechanics; in the quantum context, the subjective aspect of probability also appears in such situations, which involve state mixtures (*Gemische*, cf. [22] p. 9, [23] p. X) that are representable as statistical operators ρ but not as pure ones. However, probability in QM on this approach is found also in the *individual states* (*Zustände*, cf. [23]) as the objective aspect, representable as vectors |ψ〉 in H [23,24]. This objective contribution to the quantum probability of a measurement outcome is provided specifically by the state $|\psi \rangle =\sum_{i}|\psi_{i}\rangle$ via its complex amplitudes $\left\{c_{i}\right\}$, now known as *probability amplitudes*, as their squared magnitudes $p_{i}={\left|c_{i}\right|}^{2}$.

The quantities $\left\{p_{i}\right\}$ are understood as the probabilities of the measured system to be found to possess actual respective values of its physical properties according to the rule of Born that is elemental to the Copenhagen approach [25,26]. The measured value of a property is considered definite (actual), as opposed to indefinite (potential) [5], as discussed in great detail in [14,15]; a dynamical property of a quantum system S becomes actual with probability $p_{i}$ upon precise measurement wherein the measuring apparatus, A, must be in contact with the greater physical environment (the “the rest of the world”) and be classically describable or macroscopic (cf. [27] for a discussion of this notion and its use in the Copenhagen interpretation and elsewhere more recently). In general, some member from a set of *possible* values must occur in measurement, but the specific *actual* value measured appears randomly [28], as discussed in Section 4 and Section 5 here.

### 2.2. Quantum Indeterminacy

Another important element in the initial success of the Copenhagen approach is that it articulates well the behavior of joint probabilities appearing in the “uncertainty relations,” a manifestation of complementarity. Indeed, in Born’s view, “the factor that contributed [most]... to the speedy acceptance of [the Copenhagen] interpretation of the ψ-function was a paper by Heisenberg [29] that contained his celebrated uncertainty relations” [30]. The result of von Neumann that (sharp) observable quantities are simultaneously measurable if and only if they commute with one another and if and only if they are functions of a single observable later came to play a strong role in this understanding of such relations. It has also been shown by Pekka Lahti [31] that Heisenberg’s joint indeterminacy hypothesis—the “uncertainty principle” [29], later generalized by H. P. Robertson, [32], providing corresponding “uncertainty” relations discussed in Section 2.4 below—together with an axiomatic formulation of complementarity, when considered within the (O,S,p) framework formalized by George Mackey [19] and M. J. Maczyński [20], rigorously imply the existence of pairs of observables that cannot be jointly sharply measured. The known set of such relations has recently expanded, as shown in the next section.

The indeterminacy principle contrasts with the determinacy principle, that the magnitude of each continuous quantity is determined by a real number, as is typically assumed in classical mechanics (cf. Michael Dummett’s discussion of this principle in [33]). Note that the indeterminacy hypothesis is a statement about the associated indeterminacy of incompatible observables not algebraically commuting with each other (see Section 4 below), rather than the epistemic uncertainty regarding an independent quantity *per se*. It is, therefore, a statement to the effect that the associated properties, jointly considered, are *objectively indefinite*, as emphasized by Abner Shimony [34]; (cf. [35] Section 2.1). It is a consequence of this indefiniteness that their measured values are also not precisely predictable, that is, random in the sense explained in Section 5 below.

### 2.3. Quantum Potentia and Probability

In the Copenhagen approach, according to Heisenberg, the objective probability *p*, given by the Born rule, relates to “statements about possibilities or better tendencies (‘potentia’...)” of the system itself later to have certain actual values of measured properties [18] (p. 53). The subjective content of these probabilities is “negligible” in the pure case, i.e., where $\mathrm{tr}\rho^{2}=1$, exactly when it is a projector, which suffices for the maximal specification of the system’s actual properties; in this case, the elements of the set of quantum probabilities $\left\{p_{i}\left(O\right)\right\}$ for the outcomes $\left\{o_{i}\right\}$ in a measurement of the observable (i.e., Hermitian operator) *O* are

(1) $$p_{i}\left(O\right)=\mathrm{tr}\left(P_{i}\left(O\right)\rho \right)=\langle \psi |P_{i}\left(O\right)|\psi \rangle =\langle \psi |\phi_{i}\rangle \langle \phi_{i}|\psi \rangle \;$$

which are the squared magnitudes of the corresponding complex-valued state-vector amplitudes $\left\{c_{i}=\langle \phi_{i}|\psi \rangle \right\}$, that is, of the components of |ψ〉 in the eigenbasis $\left\{|\phi_{i}\rangle \right\}$ for *O*—the exclusivity of this form being demonstrated rigorously by Andrew Gleason [21]; see Section 4.1 below. (Here, we consider for simplicity the case of discrete properties; analogous relations hold in the continuous case.) In particular, the complex probability amplitude $c_{k}$ corresponds to the *potentia* for actualization of the specific property value $o_{k}$ upon the measurement of *O*, with $p_{k}\left(O\right)={\left|c_{k}\right|}^{2}$, something discussed further in Section 4.3 below—cf. [14]. (Very recently, the notion of quantum potentia in a sense of *res potentia* related to that of Heisenberg has been used by Ruth Kastner et al. to offer a novel analysis of quantum measurement, in combination with *res extensa*, purely physical substance, as “implicative constituents of every quantum measurement event” [36].)

Recall that the novel characteristic of the quantum probability first to be discovered (n.b. a mathematically precise treatment of its novelty in general terms has been given by Luigi Accardi in [37]), which motivates the continual reconsideration of its relationship to traditional probability, involves ostensibly disjoint events: An empirically measurable difference between the quantum mechanical probability and the classical mechanical probability of a disjunction of such events is that the associated probability is not, in general, additive in the quantum case. This is exhibited in the appearance of a particle such as an electron at a spatial location *x* in the basic double-slit experiment (cf. [38]) by passing first through a slit (Slit 1) and/or the other slit (Slit 2), which occurs with a probability $p_{12}\left(x\right)$ that is related to the probability densities of reaching *x* in the alternatives of either first passing through Slit 1, $p_{1}\left(x\right)$, or of first passing through Slit 2, $p_{2}\left(x\right)$:

(2) p12(x)∝p1(x)+p2(x).

This quantum probability is the magnitude squared of the sum $c_{12}\left(x\right)=c_{1}\left(x\right)+c_{2}\left(x\right)$ of the complex amplitudes $\left\{c_{i}\left(x\right)\right\}$ of those alternatives, rather than a simple sum of the probabilities of the two alternative situations consistent with the future event, here detection at *x*.

Thus, the quantum probabilities do not arise by direct calculation from, for example, prior probabilities of particle detection as in classical mechanics as a sum such as $p_{12}\left(x\right)\propto p_{1}\left(x\right)+p_{2}\left(x\right)$ in the situation of this double-slit experiment. (Another difference of quantum probability from classical probability is found in *joint* probabilities is discussed in Section 3 below.)

### 2.4. Quantum Interference and Dispersion

The difference between the two sorts of probability, classical and quantum, is reflected clearly in the corresponding probability density distribution in the detection plane in the double-slit experiment: There is an additional modulated “interference term” arising because the $c_{i}\left(x\right)$ are complex-valued, which precludes these probabilities from being given a straightforward Kolmogorovian representation under a single probability measure (cf. [37] and [39] p. 125, [37] for detail regarding this), so that

(3) $$p_{12}\left(x\right)\propto p_{1}\left(x\right)+p_{2}\left(x\right)+\sqrt{p_{1}\left(x\right)p_{2}\left(x\right)}\cos\left(\theta_{2}\left(x\right)-\theta_{1}\left(x\right)\right).\;$$

In this situation, there is a range of possible values for the detected position, as well as of the momentum of the system approaching it, that is, a certain dispersion of values due to its indeterminacy.

More generally, any observable given as an Hermitian operator *A* will have a dispersion ${\mathrm{Disp}}_{\rho }A=\langle {\left(A-\langle A\rangle I\right)}^{2}\rangle =\langle A^{2}\rangle -{\langle A\rangle }^{2},$ for any system in state ρ; the indeterminacy relation of Heisenberg for momentum and position relevant in this experiment was generalized by H. P. Robertson so as to apply two any two observables *A* and *B* [29,32]:

(4) $$\langle {\left(\Delta A\right)}^{2}\rangle \langle {\left(\Delta B\right)}^{2}\rangle \geq \frac{1}{4}{\left|\langle \left[A,B\right]\rangle \right|}^{2}\;$$

where the “uncertainty” of *A* for state ρ is $\Delta A\equiv \sqrt{{\mathrm{Disp}}_{\rho }A}.$ These relations are connected with single-particle interferometric complementarities, i.e., between visibility and particle path, as shown in the 1980s and 1990s; see the following section, Jaeger et al. [40] and references therein for more on the relation to interferometry, and a recent analysis of Paul Busch and Christopher Shilladay [41] for a detailed discussion of the various forms of complementarity.

Significant new developments regarding quantum probability that allow for the clearer explication of these central aspects of the Copenhagen approach are discussed in the next two sections.

## 3. Complementarity and Entanglement

The extraordinary behavior of quantum probabilities regarding compound systems due to entanglement was brought to the forefront relatively early in the history of quantum mechanics (QM) by Erwin Schrödinger [42,43] and remained the subject ongoing entirely theoretical discussions until after John Bell produced his now-famous inequality, which was subsequently rendered experimentally testable in a reformulation by John Clauser, Michael Horne, Abner Shimony, and Richard Holt (CHSH) [44] and shown to be violated in an interferometric setting in the early 1980s by Alain Aspect et al. [45].

An entanglement-related manifestation of complementarity involving joint probabilities was noted later in the 1990s: When the two-particle interference visibility is unity, the one-particle visibility is zero, and conversely as noted in the work of Marlan Scully, Berge Englert, and Herbert Walther [6] and of Shimony, Horne, and Anton Zeilinger [46]. In the mid-1990s, it was shown that the interferometric phenomena involved in the violation of CHSH inequalities obey a precise *trade-off relation*, later experimentally verified in the 2000s [40,47,48], over a full range of different experimental arrangements. This is also related to the fact that entanglement can be understood as an instance of the uncertainty of quantum properties, cf. [41]. In particular, it was found by Jaeger and Shimony that there is a general quantum interferometric complementarity relation between single-system interference visibility, $v_{1}$, and compound-system interference visibility, $v_{12}$, for pairs of two-level systems, further illustrating the surprising nature of quantum correlations exhibited in two-particle interference due to the presence of entanglement [47], as first verified at the Boston University in 2001 [48].

This novel exhibition of complementarity can be understood concretely in terms of the washing out of photon self-interference due to indeterminacy in the initial direction of individual particles in a *doubled* discrete (Mach–Zehnder, MZ) two-“slit” arrangement with a source of particle pairs at center simultaneously feeding *two* Mach–Zehnder interferometers, symmetrically oriented, with one particle moving in one interferometer involving a generalized beamsplitter (transducer) at left and similarly for the other particle moving in a second such interferometer at right (see [47] for figures). Consider two particles *A* and *B* in this arrangement, with *A* taken to be that in beams 0 and/or 1, and similarly for particle *B* (but indicated by primes below). Let each particle pair of the ensemble involved be produced by the centrally located source in a possibly entangled two-particle pure state $|\Theta \rangle =\gamma_{1}{|0\rangle }_{A}|0^{\prime }\rangle_{B}+\gamma_{2}{|0\rangle }_{A}|1^{\prime }\rangle_{B}+\gamma_{3}{|1\rangle }_{A}|0^{\prime }\rangle_{B}+\gamma_{4}{|1\rangle }_{A}{|1^{\prime }\rangle }_{B},$with $\gamma_{i}\in C$ such that $|\gamma_{1}{|}^{2}+|\gamma_{2}{|}^{2}+|\gamma_{3}{|}^{2}+{\left|\gamma_{4}\right|}^{2}=1,$${|0\rangle }_{A}$ and ${|1\rangle }_{A}$ being basis vectors in the Hilbert space $H_{A}$ of the first particle corresponding to the propagation of *A* to the left in beams 0 and 1 and $|0^{\prime }\rangle_{B}$ and $|1^{\prime }\rangle_{B}$ being similar vectors in space $H_{B}$ of *B* moving to the right.

Let beams 0 and 1 be brought together at a transducer, $T_{A}$ (inducing a general unitary transformation in the state space, not only a phase-shift+reflection/transmission as in a simple MZ apparatus), feeding two output beams, an upper *U* and lower *L* beam in the MZ interferometer at left, and let similar beams in the other interferometer to the right be brought together into another transducer, $T_{B}$ (inducing a similar unitary transformation in the other particle’s state space), that feeds two corresponding output beams $U^{\prime }$ and $L^{\prime }$; the joint, local operation of this pair of transducers is described by the general pair of local unitary operations induced by them separately: $T=T_{A}⊗T_{B}.$ As these transducers $T_{A}$ and $T_{B}$ are varied, the probabilities $P(UU^{\prime })$ of coincidence detection in beams *U* and $U^{\prime }$, and $P(UL^{\prime })$, $P(UL^{\prime })$, and $P(LL^{\prime })$, as well as single-detection probabilities $P\left(U\right),P\left(L\right),P\left(U^{\prime }\right)$, and $P(L^{\prime })$—corresponding to particle coincidence detection and single detection rates, respectively, in the output beams of the pair of interferometer—are modulated.

The corresponding visibilities of interference were found to obey trade-off relations, quantifying their complementaries. As *T* is varied over the full range of parameters for the two general local unitary transformations involved, continuously altering the apparatus, the one-particle interferometric fringe visibility $V_{i}$ (i=A,B) is found from the maximum and minimum probabilities of detection:

(5) $$V_{i}=\frac{{\left[P\left(Y\right)\right]}_{\max}-{\left[P\left(Y\right)\right]}_{\min}}{{\left[P\left(Y\right)\right]}_{\max}+{\left[P\left(Y\right)\right]}_{\min}}\;,$$

where Y=U,L. $V_{12}$, the two-particle interferometric visibility in the sense of variations of detection probability as the *T* is changed, is similarly calculable from the probabilities $P(YY^{\prime })$ of occupation of the joint-paths $YY^{\prime }$, generalizing the case of the single paths *Y*. For example,

(6) $$V_{12}=\frac{{\left[\bar{P}\left(UU^{\prime }\right)\right]}_{\max}-{\left[\bar{P}\left(UU^{\prime }\right)\right]}_{\min}}{{\left[\bar{P}\left(UU^{\prime }\right)\right]}_{\max}+{\left[\bar{P}\left(UU^{\prime }\right)\right]}_{\min}}\;$$

where $\bar{P}\left(UU^{\prime }\right)=P\left(UU^{\prime }\right)-P\left(U\right)P\left(U^{\prime }\right)+\frac{1}{4}$ represents *nonaccidental* coincidence probabilities; likewise for the three other possible path pairs $YY^{\prime }$ [47].

When the two systems, *A* and *B*, are entangled, one has the non-factoring joint probability

(7) $$P\left(UU^{\prime }\right)\neq P\left(U\right)P\left(U^{\prime }\right),\;$$

as do the other joint probabilities $P(UL^{\prime })$, $P(LU^{\prime })$, and $P(LL^{\prime })$; the extraordinarily highly correlated behavior of particles *A* and *B* arises due to entanglement, and one finds that a strong complementarity trade-off relation, taking the form of an equality [47], holds for all |Θ〉, namely,

(8) $$V_{12}^{2}+V_{A}^{2}=1\;;V_{12}^{2}+V_{B}^{2}=1\;;$$

this was subsequently experimentally confirmed by Bahaa Saleh and associates at Boston University [48,49]. This explicitly demonstrates precise quantum complementarity involving entangled systems of the sort violating the Bell and CHSH inequalities.

## 4. Quantum Measurement

In the Copenhagen approach to quantum mechanics, “the behaviour of the measuring apparatus must be capable of being registered as something actual…if the measuring apparatus is to be used as a measuring instrument at all…the connection with the external world is…necessary” [5] (p. 27); any fully quantum A and S alone would become entangled, something Don Howard has argued Bohr was already noting in 1927 [13], and neither S nor A+S can be considered closed *during measurement*. Indeed, in a fully quantum formal treatment of such a process, the apparatus would itself have to be measured in order to provide an outcome, and so on. In this approach, a fully formal quantum mechanical treatment of the measurement process utilizing a closed system description or without the use of classical descriptions for at least some elements of the measurement process is considered impossible—quantum mechanics is literally incapable of being used to *account for* all details of the process (n.b.: the adjective sometimes used in relation to this is often translated as *uncontrollable* in English, but is better translated as *unaccountable-for*.) Moreover, the objective, probabilistic aspect of quantum state evolution invoked upon actualization is considered irreducible to any amount of ignorance that might be removed using the theory alone, as mentioned in Section 2.

In the Copenhagen approach, any system S, as well as the joint system of S and any other quantum piece of apparatus A thought of as distinct from A, is an open system while being measured, because it must be coupled to the larger world to provide an actual measurement record. A coupling of quantum system S to *classically describable* devices—Heisenberg’s *recording system plus the rest of the world*, which physically intervene when using an apparatus in such a way that one among a set of differing outcomes can appear on the resulting record—is held to characterize measurement, and this coupling gives rise to a probabilistic, indeterministic state change (corresponding to the actualization of the system property) that relates to the actual value in a prescribed way (described by the EE link, see Section 4.2, below). Actualization (discussed in Section 4.3) *begins with the coupling* of the system under measurement to the classically describable observational apparatus in the world, which is physically designed (based on rigorous testing for reliability) to elicit an outcome, and *ends upon decoupling*, leaving a classically describable physical record of the outcome.

If one does consider, *contra* the views of Bohr and Heisenberg, a fully quantum mechanical closed system treatment of measurement, the behavior of the joint system A+S, or a larger chain of interactions systems (see below), this might be expected to suffice for the description of measurement. Such a treatment is often given as follows; cf. e.g., [17], [50] (Section 2.4). One prepares system S via a series of physical interactions, such as filtering, in some well-defined quantum state |η〉, after which it is arranged to interact with a measurement apparatus, A. This apparatus, after being similarly arranged to be in a fiducial initial state $|\chi_{0}\rangle$, would be required to enter a final state corresponding to the value of a pointer property *Z*, which must be correlated with the value of the measured property (non-degenerate observable) *E* of the system. We may consider, for simplicity, a discrete measured property

$$E=\sum_{i}e_{i}|\psi_{i}\rangle \langle \psi_{i}|$$

where $\left\{|\psi_{i}\rangle \right\}$ is a countable orthonormal basis for the system Hilbert space H corresponding to its eigenvalues $\left\{e_{i}\right\}$. Another typical requirement for such a measurement is that a “calibration condition” be satisfied, namely, that if a measured property is real, then its value must be exhibited properly, unambiguously, and with certainty: if system S is an eigenstate $|\psi_{k}\rangle$ of *E*, then the state of apparatus A after the interaction of the two is an eigenstate of *Z* (with an eigenbasis $\left\{|\phi_{i}\rangle \right\}$ associated with pointer readings $z_{i}$), which serves to indicate the specific value of *E* present, the free-Hamiltonian function contribution to the evolution of the system being considered negligible relative to that of the measurement interaction. For quantum observables, the calibration condition generally takes the form of a probability reproducibility condition, namely, that a probability measure exists for a property be transcribed onto that of the corresponding apparatus pointer property. Finally, registration of the measured property by the measurement apparatus is taken to include the physical reading out of the registered value.

If one formally considers an entire chain of interacting objects connecting the system S up as far as physically conceivable, to the brain of an experimenter, for example X, Y,…, in the environment in addition to the original measurement system S and the experimenter’s apparatus A—such as focusing elements, counter or counters, various cables, a computer, output display, etc.—a good measurement would involve all these becoming correlated in their properties for the measurement outcome to be physically indicated. Under the Schrödinger state evolution, which is unitary, upon completion of the measurement interactions, one would then find

(9) $$|\Psi \rangle =\sum c_{i}|s_{i}\rangle |a_{i}\rangle |x_{i}\rangle |y_{i}\rangle \ldots \;,$$

with $\left\{s_{i}\right\}$, $\left\{a_{i}\right\}$, $\left\{x_{i}\right\}$, $\left\{y_{i}\right\}$ etc. as the Hilbert space eigenbases for S, A, X, Y,*…*, respectively. The result of considering all physical systems involved entirely quantum mechanically is simply a regress backward from the prepared state, which presents and indefinite value of the quantity to be observed.

Heisenberg had already engaged this difficulty early on (in 1935 [51], cf. [52]) in a Copenhagenist spirit by insisting on a bipartite division of a set of different systems involved, only one of which is to be considered in any one analysis among all those possible, one for each way of making a bipartite division of the above chain, and considering only *one side of the division* quantum mechanically, as described below, cf. [51]. Again, for him, consideration of the entire measurement chain—or even simply the system and portion of apparatus in direct contact with it—as a full accounting of the measurement process as described within the state-vector formalism, as done in the above, without truncation, is an inappropriate use of the quantum formalism, the proper role of which is *to make predictions of measurement outcomes*; the only plausible use of the quantum formalism for the purposes of symbolizing a measurement process requires the introduction of a cut or split (*Schnitt*) between what is considered the measured portion of this chain of systems on the side including the entity S and the remainder, considered then to be a single, classically describable measuring system.

Notably, a change in the location of the cut makes no difference to the statistics obtained for the purposes of prediction. This formal description is strictly speaking only a symbolic description of the elements of the world involved. Heisenberg also imposed an important condition restricting this location: “The claim … that it is indifferent at which location the cut between the parts of the system to be treated quantum mechanically and the classical measuring devices should be drawn, should thus be made more precise in the sense that this cut may indeed be shifted arbitrarily far in the direction of the observer in the region that is otherwise described according to the laws of classical physics; but that this cut cannot be shifted arbitrarily in the direction of the atomic system. Rather, there are physical systems—and all atomic systems belong among these—that the classical concepts are unsuitable to describe, and whose behaviour can therefore be expressed correctly only in the language of wavefunctions” [51], cf.[53].

In this way, the chain of statistical correlations appearing in the formal representation of the state |Ψ〉 is considered to be cut in two—into a system S${}^{\prime }$ and the remainder A${}^{\prime }$—somewhere along this chain of interacting systems, with subsystems to one side of the cut collectively considered the system to be measured: S${}^{\prime }$ subsumes S together with all other subsystems left of the cut, and A${}^{\prime }$(=A + W) is the collective of those systems right of the cut, that is, the “apparatus plus the rest of the world” W. Thus, A${}^{\prime }$, is removed from the quantum-physical description used to make predictions relating to outcomes, with the cut always being made *somewhere* within S–X–Y–⋯–A–W, where a classical description is possible for the entire portion including the recording system. The actualization of potentia requires an interaction of S${}^{\prime }$ (the size of S or larger) with the classically describable measuring apparatus, itself in interaction with the rest of the world. Formally, the change of state of the measured system involved is sometimes said to be “projected” to the appropriate component of the initially prepared system state, that is to be attributed by the eigenvalue-eigenstate link (discussed below in Section 4.2). Such a projection involves a change of state differing from the unitary evolution predicted when using the Schrödinger law of motion alone, for any non-trivial measurement.

It should be noted, however, that the Copenhagen approach to measurement can be criticized for not offering, indeed, for *denying the possibility of* a complete, closed system description of the measurement process or, for that matter, even precisely specifying the conditions under which measurement will occur, for example, due to the unclear boundary between the classical and the quantum realms, by its reliance on the requirements of the use of a macroscopic apparatus and the production of classically describable records of reliable measuring instruments not precisely characterizable by quantum mechanics. For this reason, Heisenberg’s appeal to actualization (discussed in Section 4.3) can be considered an incomplete quantum mechanical treatment. Moreover, descriptions of the sort given by Heisenberg in the above have been criticized for conflating measurement with state preparation, as Henry Margenau did already in 1936 [54].

Let us turn now to Heisenberg’s indeterminacy relations. In the presentation of the indeterminacy relations in Heisenberg’s 1929 Chicago lectures, published as *The Physical Principles of the Quantum Theory* [29], in which he spoke only of the *Der Kopenhagener* Geist *der Quantentheorie* rather than a full *interpretation*, the indeterminacy relations were, strictly speaking, only symbolic in nature and in the process of being generalized (beginning with work by H. P. Robertson [32] discussed in Section 2 above). These relations were placed on a firm mathematical grounding soon thereafter in the Hilbert-space formalism; cf. Section 2.4. Recently, notably since the final explication of the Copenhagen approach by Heisenberg in the 1950s [5], these relations have been analyzed, extended, and clarifed via the notion of *unsharpness* in a way that captures the notion of quantum indeterminacy more efficaciously.

### 4.1. Unsharpness

The maximally specified state of a quantum system relative to an observable *O* in the Copenhagen approach can be given as a projector $\rho_{\mathrm{pure}}=\left|\psi \rangle \langle \psi \right|$ appearing in the spectral decomposition of an observable *O*, as discussed in Section 2.1; cf. [2,55]. Nonetheless, in addition to measurements corresponding to such operators, *unsharp measurements* have also been formalized as the class of quantum operations that are described by (normalized) *positive-operator-valued measures* (POVMs) developed by Günther Ludwig, Karl Kraus, Busch, and Lahti [17,56,57].

Given a nonempty set *S* and a σ-algebra Σ of its subsets $X_{m}$, a POVM *E* is a collection of operators $\left\{E\left(X_{m}\right)\right\}$ satisfying the conditions: (i) *Positivity*—$E\left(X_{m}\right)\geq E\left(\emptyset \right)$, for all $X_{m}\in \Sigma$; (ii) *Additivity*—for all countable sequences of disjoint sets $X_{m}$ in Σ, $E\left(\cup_{m}X_{m}\right)=\sum_{m}E\left(X_{m}\right)$; (iii) *Completeness*—E(S)=I. If the *value space* (S,Σ) of a POVM *E* is a subspace of the real Borel space (R,B(R)), then *E* provides a unique Hermitian operator on H, namely $\int_{R}\mathrm{Id}\;dE,$ where Id is the identity map. The positive operators $E(X_{m})$ in the range of a POVM are referred to as *effects* E(H)={A∈L(H):O≤A≤I}, the expectation values of which provide the quantum probabilities.

Given an effect *A*, one can define properties in general by the following set of conditions. (i) There exists a property $A^{⊥}$; (ii) there exist states ρ and $\rho^{\prime }$ such that both $\mathrm{tr}\left(A\rho \right)>\frac{1}{2}$ and $\mathrm{tr}\left(A\rho^{\prime }\right)>\frac{1}{2}$; (iii) if *A* is regular, for any effect *B* below *A* and $A^{⊥}$, $2B\leq A+A^{⊥}=I$, where a *regular effect* is an effect with spectrum both above and below $\frac{1}{2}$. Thus, the set of properties is $E_{p}\left(H\right)=\left\{A\in E\left(H\right)|A≰\frac{1}{2}I,A≱\frac{1}{2}I\right\}\cup \left\{O,I\right\}$; the set of *unsharp properties* is $E_{u}\left(H\right)\equiv E{\left(H\right)}_{p}/L\left(H\right).$ A POVM is an *unsharp observable* if there exists an unsharp property in its range [2]. The POVMs are the natural correspondents in the operator space of quantum mechanics of standard probability measures and thereby make precise the notion of indeterminacy in the Hilbert-space setting.

The probability of a given outcome *m* upon a (generalized) measurement on a system in a pure state P(|ψ〉) is given by

(10) $$p\left(m\right)=\langle \psi |E\left(X_{m}\right)|\psi \rangle =\mathrm{tr}\left((|\psi \rangle \langle \psi |)E\left(X_{m}\right)\right)\;;$$

cf. Equation (1), which holds for the case of sharp measurement. The effects form a convex subset of the space of linear operators on L(H) on the system Hilbert space, the extremal elements of this subset being the projectors $\left\{P_{i}\right\}$. A collection of effects is said to be *coexistent* if the union of their ranges is contained within the range of a POVM. Any two quantum observables $E_{1}$ and $E_{2}$ are representable as sharp measures on (R,B(R)) exactly when $[E_{1},E_{2}]=O$, following from the results of von Neumann for Hermitian operators [23] mentioned in Section 2 above; coexistent observables are thus those that can be *measured simultaneously in a common measurement arrangement*, and when two observables are coexistent, there exists an observable, the statistics of which contain those of both observables, the *joint observable*. Typically, the two observables are recoverable as marginals of a joint distribution on the product of the corresponding two outcome spaces.

The introduction of unsharpness allows one to circumvent the requirement of commutativity of jointly measurable properties, which captures only the extremes of complementarity, by including the unsharp properties, and enables a continuous range of complementarity to be captured in the Hilbert space formalism. For POVMs, commutativity remains sufficient but is not necessary for the coexistence of effects (cf. [2]). It has been shown that “smeared versions” of two noncommuting observables can still have a joint observable. For example, the operators $F=\{\frac{1}{2}\left(I\pm f\sigma_{z}\right)\}$ and $G=\{\frac{1}{2}\left(I\pm g\sigma_{z}\right)\}$ have a joint observable precisely when $f^{2}+g^{2}\leq 1$. Therefore, as a requirement for this pair to be jointly observable, the magnitudes |f|,|g| (their degrees of unsharpness) must be complementary, in accordance with this trade-off relation, as demonstrated by Busch and Shilladay [41].

The introduction of POV measures and unsharpness have thus helped make indeterminacy and mathematical complementarity more precise by exploiting the Hilbert-space setting; cf. [41] for further detail on the intertwined connection and contrast between those two notions and more detail on their role in understanding joint measurability of properties in experimental situations, such as the single-particle Mach-Zehnder interferometry, not discussed here.

### 4.2. Linking Actual and Possible Values

In the Copenhagen approach to quantum mechanics, the state of a measured system is related to the actual values obtained in measurement by what has come to be known as the eigenvalue-eigenstate (EE) link. The essence of the EE link was first introduced by Heisenberg and then used by others including Dirac in 1930 and after; cf. the careful explanation of this by Marian Gilton [58]. In the 1930 version of Dirac’s *Quantum mechanics*, one finds the following:

“If a state $\psi_{r}$ and an observable α are such that, when an observation is made of the observable with the system in this state the result is certain to be the number *a*, we assume this information can be expressed by the equation

(11) $$\alpha \psi_{r}=a\psi_{r}$$

Conversely, when an equation of this type is given, we assume it has the physical meaning that a measurement of the observable α with the system in state $\psi_{r}$ will certainly give for result the number *a* or that the observable α has the value *a* for the state $\psi_{r}$, to use a classical way of speaking, which is permissible in this case”

[24] (p. 30).

In 1958, in the 4th edition of his classic textbook *Quantum Mechanics*, which appeared after Heisenberg’s article “The Development of the Interpretation of Quantum Mechanics,” one finds the EE link connected explicitly with probability.

“The expression that an observable ‘has a particular value’ for a particular state is permissible in quantum mechanics in the special case when a measurement of the observable is certain to lead to the particular value, so that the state is in an eigenstate of the observable … In the general case we cannot speak of an observable having a value for a particular state, but we can speak of its having an average value for the state. We can go further and speak of the probability of its having any specified value for the state, meaning the probability of this specified value being obtained when one makes a measurement of the observable.”

[59] (p. 253)

### 4.3. Causation, Possibility, and Actuality

Like other sorts of probability, quantum probability can be viewed as the “graded possibility” of the occurrence of events, as first suggested by Leibniz [60,61]. Moreover, in the Copenhagen approach to quantum mechanics, unlike others, possibility plays a fundamental role in relating theory to experiment. This was explicitly indicated by Heisenberg in his invocation of an aspect of Aristotle’s theory of causation, wherein possibility appears prominently in relation to all phenomena: “…in modern physics the concept of possibility, that played such a decisive role in Aristotle’s philosophy, has moved again into a central place” [62] (p. 298).

As pointed out in Section 2 above, the isolated quantum system, described by a state-vector |ψ〉∈H, “no longer contains features connected with the observer’s knowledge…it is also completely abstract …and the representation becomes a part of the description of Nature only by being linked to the question of how real or possible experiments will result” [5] (p. 26). The objective aspect of probability is that of quantifying the likelihood $|c_{i}{|}^{2}$ of the appearance of each value among any set of possible measurement outcomes as the *actual* result in the actualization of the potential physical state which occurs upon measurement, which according to Heisenberg is independent of any subjectivity: “the transition from the ‘possible’ to the ‘actual’ takes place as soon as the interaction between the object and the measuring device, and thereby with the rest of the world, has come into play; it is not connected with the act of registration of the result in the mind of the observer” [18] (pp. 54–55).

Heisenberg explained the objective character of this registration process as follows.“Of course, the introduction of the observer must not be misunderstood to imply that some kind of subjective features are to be brought into the description of Nature. The observer has rather only the function of registering decisions, i.e., processes in space and time, and it does not matter whether the observer is an apparatus or a human being; but the registration, i.e., the transition from the possible to the actual, is absolutely necessary here, and cannot be omitted from the interpretation of the quantum theory. It must also be pointed out that in this respect the Copenhagen interpretation of quantum theory is in no way positivistic. For whereas positivism is based on sensual perceptions of the observer as elements of reality, the Copenhagen interpretation regards things and processes which are describable in terms of classical concepts, i.e., the actual, as the foundation of any physical interpretation.” [5] (p. 22). Thus, Heisenberg neither requires nor refers to the mind, the brain, or human knowledge for the *actualization* of potential values, which appear in successful measurements as actual values of an observable inferrable from a resulting classically describable record in accordance with the EE-link; only the interaction of the measured system with the greater world in a way so as to produce such a classical record is required. It is such a record from which the mind could later acquire knowledge if the recorded, classically describable measurement outcome is later attended to.

Measurement of a quantum system precludes the state change that would otherwise occur were it to remain isolated, that is, the time-evolution according to the Schrödinger law of motion. It is in this way that, in the Copenhagen approach to QM, the possible becomes the actual and can be recorded, according to Heisenberg—a way not necessarily captured by the evolution dictated by the law of motion; it is often said that it is at this point that *causation* fails in QM—that it becomes acausal. However, this non-deterministic change of state-vector evolution arises precisely with the intervention of the measuring apparatus and the rest of the world participating in the production of a classical record of the outcome as its *cause*.

Hence, the Copenhagen approach to non-relativistic quantum mechanics presented by Heisenberg is not one in which there is a genuine *lack* of causation; instead, there is a form of *Aristotelian causation* that is not captured by the fundamental law of motion, which only governs closed systems not being measured—in particular, it is the form Aristotle calls *chance* causation, as I have argued in [14,15]: In the actualization of potentiality, there is a single chance occurrence that lies within the set of possible occurrences for the system in question according to the characteristics of its Hilbert-space description, with a measured value capable of being recorded on a system that is also classically describable but unpredictable [5]. It is in this way that the Copenhagen approach remains, in a specific sense, causative. This approach to quantum probability, where it fundamentally involves possibility, has recently been connected with logic in relation to the possible experience to be gained through measurement, as shown in the next subsection.

### 4.4. Logic and Indeterminacy

In the Copenhagen approach, the quantum state is taken to characterize as completely as possible the system to which it is attributed. The changes in the world occurring in any measurement are changes of values of quantities that were theretofore potential, that is, only possibly possessed, in accordance with the EE link connecting possible and actual observed values of observables. Empirically, the probabilities of these various outcomes to occur accord with the likelihoods of obtaining the set of possible outcomes for measurements in the future correspond formally to the Hermitian operator *O*, in measurements on collections of identically prepared systems described by the state |ψ〉 with the resulting measurement record being classically describable when necessary for their readout. These probabilities have been connected to logic; cf. [1] (Chapter 8). Indeed, the results of the early work of von Neumann and Birkhoff associates propositions to quantum Hilbert (sub)spaces [63] and the field of quantum logic that arose from that pioneering work; cf. [64] for a general summary of later developments.

#### 4.4.1. Completeness of the Quantum State Description

Whether the quantum state was indeed complete, despite the apparent incompatibility of this with the attribution of precise values to *all* observable quantities, remained unclear until the work of Simon Kochen and Ernst Specker in 1967 [65]. Their theorem now known as the Kochen-Specker theorem precludes a consistent truth-valuation from being given to the propositions identified by von Neumann and Birkhoff, which is what is required to satisfy the corresponding value-definiteness thesis, namely, (i) that each and every physical magnitude have a definite value at all times (see Alan Stairs’ valuable discussion [66]) and (ii) that measurements reveal those preexisting precise values; cf. [50] (Section 2.3) for a discussion of related issues. The value-definiteness condition can be stated more formally: Each proposition regarding the system, of the form “O∈Δ,” where *O* describes the physical magnitude and Δ is a Borel subset of the real numbers, is given a definite value 0 or 1.

The work of Kochen and Specker followed a very general theorem of Andrew Gleason [21]: All probability measures that can be defined on the lattice of quantum propositions from the quantum statistical operators, that is all quantum probabilities, are of the form $p\left(P_{i}\right)=\mathrm{tr}\left(\rho P_{i}\right)$, for some statistical operator ρ on Hilbert space H, for all H of dimension greater than two. This result demonstrates that every probability measure over the set of quantum state projectors is one from a quantum state ρ on the H attributed to the system in question; the trace measure $\mathrm{tr}(\rho P_{i})$ assigns to each projector $P_{i}$ the dimension of its range, which can then be normalized by the dimension of the (finite-*d*) H. It can, therefore, be obtained by taking ρ to be the maximally mixed state on H. This shows that the only natural generalization of Kolmogorov probability functions of the sort used in quantum mechanics is exactly that of the Hilbert-space formulation of quantum mechanics. The values corresponding to orthogonal projectors thus obey a Born-type rule for the assignment of probabilities.

States for which definite truth-values could be attributed to all observables are the so-called dispersion-free states, states for which projectors take expectation values of only either 0 or 1 under the above mapping. Following a presentation first given by Bell, we can relate this to Gleason’s work [67]. The condition $\sum_{i}\langle P(|\phi_{i}\rangle )\rangle =1$ implies that both 0 and 1 occur because (1) there are no other possible values for satisfying the condition and (2) neither alone suffices. However, then, there must be arbitrarily close pairs |ψ〉,|ϕ〉 having different expectation values, 0 and 1 respectively; however, such pairs cannot be arbitrarily close, by the above lemma. Hence, there can be *no* dispersion-free states providing the statistics of quantum statistics. Accordingly, no variables parameterizing dispersion-free probability measures can exist for systems having H [67]. The set of quantum states is therefore complete, because it provides the probability measures definable on the quantum propositional lattice.

Consider the complete set of Hermitian self-adjoint operators for the set of quantum states describing a system attributed a Hilbert space with dimension d>2, constraining their algebra to reflect the values assigned, and take the assignment of the values of real numbers to the quantum operators to reflect corresponding properties of the system. In this setting, the Kochen–Specker theorem demonstrates the impossibility of such an assignment for a finite sublattice of quantum propositions [65]. Take a *value function*, $v_{\psi }$ connecting an observable *O* to a value of a physical magnitude O when a system is in a state ψ, that is, $v_{\psi }:O\to R$. Define F(O) to be the value associated with F(O) for all functions *F* with a one-to-one, onto mapping from values of O to *O*. Taking $v_{\psi }\left(F\left(O\right)\right)=F\left(v_{\psi }\left(O\right)\right)$ has the consequence that $v_{\psi }$ is additive and multiplicative on commuting operators with the consequence that $v_{\psi }\left(I\right)$ for all states ψ as long as there is at least one magnitude O for which $v_{\psi }\left(O\right)=0$ (*cf.* [68], pp. 191–192). Another consequence of this is that $v_{\psi }\left(P_{i}\right)$ must be either 0 or 1 for all propositions $P_{i}$, which have corresponding projectors $P_{i}$. Thus, if one considers a resolution of the identity into a set of projectors $\left\{P_{i}\right\}$, that is, this set is such that $\sum_{i}P_{i}=I$, in an interpretation of quantum properties where one and only one of the corresponding magnitudes P${}_{i}$ can take the value 1, no such function exists except for an overly restricted class of properties.

The results of Gleason as well as Kochen and Specker thus support the basic Copenhagen assumption that the quantum state is complete. Note also that the Kochen–Specker result can be extended to general von Neumann algebras, as shown by Andreas Doring et al. in [69], with implications quantum and generalized probabilistic models, as noted by Federico Holik et al. in [70].

#### 4.4.2. Logical Quantum Indeterminacy Relations

More recent work of Itamar Pitowsky has shown that this connection can be placed in the context of indeterminacy to provide a new class of trade-off relations exhibiting complementarity in logical context. In his investigation, Pitowsky began by noting that George Boole, in developing his conception of probability, identified necessary and sufficient conditions for a set of rational numbers $p_{1},p_{2},...,p_{n}$ to represent properly the probabilities, considered (relative) frequencies, of the occurrence of a set of *n* logically connected events $E_{1},\ldots E_{n}$ [71] $p_{i}=\mathrm{prob}\left(E_{i}\right)\;\;i=1,2,...,n$ to express what Boole called “conditions of possible experience” [72]. These conditions are either linear inequalities or equalities in $p_{1},p_{2},\ldots ,p_{n}$; if the events under consideration are entirely independent, then the fractions corresponding to probabilities might be constrained only by the conditions $p_{i}\geq 0,p_{i}\leq 1\;,$ but the expression for sets within possible experience must take the simple form

(12) $$a+\sum_{i}^{N}a_{i}p_{i}\geq 0\;$$

where $a,a_{i}$ are constants involving the logical relations constraining them [72].

This set of conditions on probabilities lie within *n*-dimensional polytopes in the case of probabilities of correlation, the convex hull of a finite number of points in $R^{d}$, that is, the set of all convex combinations of its points [73]. Any violation of these conditions is manifested geometrically by the location of points (corresponding to probabilities) outside of the polytope dictated by them. The conditions on possible experience can then be methodically constructed from the logical relations among sets of possible events. Take, for example, a pair of events $E_{1},E_{2}$ having relative frequencies $p_{1},p_{2}$, again taking $p_{12}$ to denote the frequency of the *joint event* $E_{1}\cap E_{2}$. Being probabilities, these numbers have the relations: $p_{1}\geq p_{12},\;\;p_{2}\geq p_{12},\;\;p_{12}\geq 0$. The frequency of the disjoint event $E_{1}\cup E_{2}$ is then $p_{1}+p_{2}-p_{12}$ with

(13) $$p_{1}+p_{2}-p_{12}\leq 1\;.$$

One then has a corresponding three-dimensional space of vectors $\left(p_{1},p_{2},p_{12}\right)$ that can be viewed as a convex polytope with vertices (0,0,0),(1,0,0),(0,1,0), and (1,1,1). Pitowksy considered a set of measurements known to have as outcomes 0 and 1, such as the measurements on a squared value $S_{i}^{2}$ of the component of spin along orthogonal spatial directions for a spin-1 system.

In this case, the basic operators $S_{i}$ do not commute and so cannot be precisely measured simultaneously, while their squares do, cf. Pitowsky’s [74]. Their sum $S^{2}=2I$, where I is the identity, so that in a simultaneous measurement of these spin-squared operators, one and only one of these observables will have the value 0, while the others take value 1. This illustration of the general situation corresponds to measurements with a triple of possible outcomes. Let the events that appear in more than one measurement be written as $E_{i}$, and let those that appear in only one triple be $F_{i}$. Suppose the noncontextuality of probability—the requirement that probability assignments do not depend on the outcomes of measurements of other observables that might be measured at the same time, cf. [75,76], and assign the same probability to each event above in all cases. Given that the probabilities in each triple of possible outcomes must also sum to one, one then finds

(14) $$\begin{array}{ccc} p\left(E_{1}\right)+p\left(E_{2}\right)+p\left(F_{2}\right) & = & 1 \end{array}$$

(15) $$\begin{array}{ccc} p\left(E_{1}\right)+p\left(E_{3}\right)+p\left(F_{3}\right) & = & 1 \end{array}$$

(16) $$\begin{array}{ccc} p\left(E_{2}\right)+p\left(E_{4}\right)+p\left(E_{6}\right) & = & 1 \end{array}$$

(17) $$\begin{array}{ccc} p\left(E_{3}\right)+p\left(E_{5}\right)+p\left(E_{7}\right) & = & 1 \end{array}$$

(18) $$\begin{array}{ccc} p\left(E_{6}\right)+p\left(E_{7}\right)+p\left(F_{1}\right) & = & 1 \end{array}$$

(19) $$\begin{array}{ccc} p\left(E_{4}\right)+p\left(E_{8}\right)+p\left(F_{4}\right) & = & 1 \end{array}$$

(20) $$\begin{array}{ccc} p\left(E_{5}\right)+p\left(E_{8}\right)+p\left(F_{5}\right) & = & 1. \end{array}$$

These requirements on probability then imply trade-off inequalities expressing complementarity, for example,

(21) $$p\left(E_{1}\right)+p\left(E_{8}\right)\leq 3/2\;.$$

One of the two outcomes $E_{1}$ and $E_{8}$—which cannot arise as alternative outcomes of the same measurement—becomes more certain, that is, there is an increased probability of occurrence. The other outcome becomes less certain, with a decreased probability of occurrence. This is a logic-based indeterminacy relation quantitatively expressing that the likelihoods of positive results in alternative measurement arrangements are complementary quantities [74].

Here again, a novel sort of indeterminacy relation was obtained and given in the clear mathematical form of a trade-off relation—this in addition to the others introduced and developed after the advent of the Copenhagen approach. This new sort of relation arises from the consideration of a collection of sets of alternative events in Boolean logic within single measurements, with the two events involved resulting from a collection of such measurements as outcomes of different measurements, as discussed in [77]. Like those discussed in the previous section, this result furthers the significance the Copenhagen approach’s notions of complementarity and indeterminacy by revealing their appearance beyond its original mathematical locus, further demonstrating its fundamental significance.

## 5. Indeterminism and Randomness

Classical physics has most often been thought to be deterministic in the following sense, introduced by Laplace in the following statement. “We ought…to regard the present state of the universe as the effect of its anterior state and as the cause of the one which is to follow. Given for one instant an intelligence which could comprehend all the forces by which nature is animated and the respective situation of the beings who compose it—an intelligence sufficiently vast to submit these data to analysis—it would embrace in the same formula the movements of the greatest bodies of the universe and those of the lightest atom; for it, nothing would be uncertain, and the future, as well as the past, would be present to its eyes.” [78]. Under the conditions set out in this statement of the notion, perfect predictions and retrodictions regarding the behavior of individual physical systems are possible, in principle.

The determination of future (and past) events involved here is identified with *in-principle predictability* under the assumption that unlimited resources are available to the predictor, P: It involves in its application the existence of an intelligence with unlimited capabilities, both physical and computational. However, this is something beyond the scope of the practice of physics from within the universe: Finite beings—with finiteness assessed via the physical and/or cognitive resources at their disposal—are inherently limited in their ability to predict physical events, given that prediction requires computational resources; in any application of physical theory, such as prediction, any finite agent can exploit only *finite* physical resources for this purpose.

### 5.1. Predictability

Although the contraints on the resources of finite beings do not always present difficulties for precisely predicting a given future event among a given finite set of events in classical mechanics, even unlimited resources do not suffice for *quantum mechanics* to be considered a deterministic theory according to the above classic definition and that even when one among a *finite* set of alternatives need be distinguished. An alternative, more physically straightforward definition of determinism applicable to quantum mechanics would therefore be superior, in particular, one more suitable to physics and independent of radical assumptions about the availability of computional and other resources. One such a definition that has been suggested is: A scientific theory is *deterministic* if and only if in that theory any two trajectories in the state space in models of systems overlap at one point do overlap at *every point*, and it is indeterministic if and only if it is not deterministic (cf. discussion in [79,80,81]).

Judging this within the (O,S,p) framework of general physics, one sees that quantum mechanics is *not* a theory supporting determinism of this latter kind *for individual, measured systems*, but at best provides precise predictions of the behavior of collections of identically prepared and subsequently measured systems: On the Copenhagen approach, quantum measurement of physical properties are understood to introduce a probabilistic change of physical state that precludes the state-trajectory overlap required by this definition. The “acausality”—as it was often called early in the history of the approach—of this quantum state evolution is responsible for the indeterminism of the states actually appearing from among those possible beforehand as the result of measurement. The states connected with measurement data are always those dictated by following the EE link rule, which offers an alternative to indeterminacy (“uncertainty”) relations for capturing the objective indefiniteness of physical properties. As explained in Section 4.3 above, the Copenhagen approach provides an Aristotelian form of causation for this process even in the presence of chance.

### 5.2. Randomness

Despite the clarity gained by the move to a trajectory-based version of determinism, predictability remains relevant to the question of the randomness of the appearance of measurement outcomes. Note that a crucial distinction exists between indeterminism and unpredictability: Predictability hinges on the total context of prediction of *P*—be that a human or another sort of cognitive agent such as an artificially intelligent robot—including the all the conditions of the experimental context. Indeterminism, in the trajectory-based definition just discussed, involves only the theoretical character of the theory involved, here QM, specifically regarding the topology of state-space trajectories, independently of whether or how they could, if ever, come to be *known*. The randomness appearing in quantum mechanics should not be identified with essentially probabilistic state evolution, that is, indeterminism, as noted by Geoffrey Hellman [82], because indeterminism is not a necessary condition for the appearance of randomness in a theory.

Randomness can be defined as maximal unpredictability, and the Copenhagen approach provides a consistent understanding of the notions of indeterminism and unpredictability matching this conception well. A notion of predictability that accords with one of the distinguishing characteristics of the approach, namely, the involvement not only of the measurement apparatus but also the large world (environment) of the measurement apparatus in the very definition of proper measurement (cf. [5,10,14]), has been recently introduced by Anthony Eagle [81], namely, this physical process definition:

“A prediction function p(S,t) takes as input the current state *S* of a system described by a theory *T* as discerned by a predictor *P* and an elapsed time *t*, and yields a temporally indexed probability distribution Pr${}_{t}$ over the space of possible states of the system. A *prediction* is a specific use of some prediction function by some predictor on some initial state $S_{0}$ and time $t_{0}$ who adopts Pr${}_{t}$ as their posterior credence function conditionally on the evidence and the theory.”

[81]

This definition of predictability when applied using the probabilities of outcomes of quantum measurements allows their random character to be explicated.

The randomness in quantum mechanics in the Copenhagen approach can be understood specifically as follows. Let *P* be any experimenter performing a measurement, let *T* be the quantum mechanics, let $|a_{0}\rangle$ be the the quantum state attributed to the pertinent system via the preparation procedure used by *P* at time $t_{0}$, and let *t* be the time elapsed between its preparation and the completion of the measurement. Predictor *P* will use the appropriate choice of quantum probability as his/her $p(|a_{0}\rangle ,t)$, according to the circumstances of the measurement it performs, that a given outcome *b* obtains, and use it in finding his posterior credence function given his entire background knowledge. In particular, one can apply the following definition of *unpredictability*: “An event *E* (at some temporal distance *t*) is unpredictable for a predictor *P* if and only if *P*’s posterior credence in *E* after conditioning on current evidence and the best prediction function available to *P* is not 1, that is, if the prediction function yields a posterior probability distribution that does not assign probability 1 to *E*” [81].

Given, in particular, that measurement results are unpredictable in this sense whenever incompatible observables are measured in succession, one then has a well defined sense of *randomness* that applies to quantum mechanics:

“An event *E* is random for a predictor *P* using theory *T* if and only if *E* is maximally unpredictable. An event *E* is maximally unpredictable for *P* and *T* if and only if the posterior probability of *E* yielded by the prediction functions that *T* makes available, conditional on current evidence, is equal to the prior probability of *E*. This also means that *P*’s posterior credence in *E*, conditional on theory and current evidence (the current state of the system), must be equal to *P*’s prior credence in *E* conditional only on theory.”

where *E* is the appearance of the eigenvalue *b* as the measurement outcome. The quantum measurement process is then seen to be random when successive measurements of non-commuting sharp observables are made: Knowledge of the outcome obtained for state preparation via measurement of given quantum observable does not provide additional information about the outcomes of measurements of a sharp observable with which it does not commute, such as when the x-spin of a spin-1/2 particle is measured just after its z-spin is measured, for example, using a Stern-Gerlach apparatus.

The general notion of randomness is as an extrinsic property of events that is dependent on properties of agents such as *P*—and, more importantly, the scientific communities they form—and the theories they use pertaining to all elements involved in their scientific activity. However, it only requires that an account of how probability influences credence need not involve an interpretation of probability that itself depends on credence, and in no way requires a *subjective* interpretation of the probability attributed by quantum mechanics; it only recognizes that *predictability* expressed via Pr${}_{t}$ is *epistemic* in character, cf. [81]. This notion is one that the reinforces the Copehagen approach: The randomness in quantum theory is evidenced in relation to measurements when two non-commuting sharp observables are measured in succession. The result of the second measurement is maximally unpredictable using the law of motion to predict it on the basis of the result of the first measurement and any other information obtained from the world external to the system.

## 6. Conclusions

Results from the investigation of the behavior of quantum probability in a range of novel circumstances after Heisenberg’s clarification of the elements of the Copenhagen interpretation in the mid-1950s were shown to clarify further the nature and identity of the forms of causation, indeterminacy, and randomness that the interpretation attributes to quantum mechanics. A number of novel trade-off relations quantifying complementarity in additional areas of mathematics and physics were reviewed and described, as was the introduction of a number of mathematical constructions making indeterminacy more precise and extending its application. Results showing that the chancy nature of the results of measurements on quantum systems, and hence of the appearance of the quantum state, can be explicated in a way that accords with the Aristotelian form of causation introduced by Heisenberg in his late explication of the Copenhagen interpretation were also reviewed. These various developments in theoretical physics, some of which also bear directly on experimental physics, that appeared subsequent to Heisenberg’s clarification were in this way shown to demonstrate the continually increasing value to physics of the Copenhagen approach to quantum mechanics.
